# Political grief and ambiguous loss in a threatened democracy: psychological distress and civic responses during Israel’s judicial reform

**DOI:** 10.3389/fpsyt.2025.1687951

**Published:** 2026-01-12

**Authors:** Einat Yehene, Shay Ohayon

**Affiliations:** 1 School of Behavioral Sciences, The Academic College of Tel Aviv-Yaffo, Tel Aviv, Israel; 2 Department of Psychology, Bar-Ilan University, Ramat-Gan, Israel

**Keywords:** ambiguous loss, political grief, political self-efficacy, civic engagement, protest

## Abstract

**Background:**

Political transitions can have profound mental health effects, yet political grief remains underexplored. Drawing on ambiguous loss and grief theory, this study examined how perceived democratic backsliding during Israel’s 2023 judicial reform affected psychological distress and civic engagement.

**Methods:**

A cross-sectional survey was conducted with 453 Israeli adults opposing the reform. Measures included political ambiguous loss (PAL), political grief (adapted PG-13 scale), psychological distress (DASS-21), political efficacy, and protest participation.

**Results:**

64.9% of participants exceeded the PG-13 threshold, indicating elevated political grief. Moderate to severe depression, stress, and anxiety were reported by 32.0%, 26.3%, and 10.8% of participants, respectively. Political grief fully mediated the relationship between PAL and distress, accounting for 41% of the variance in depression, 30% in anxiety, and 36% in stress, controlling for socio-demographic variables. Logistic regression showed that higher stress, political efficacy, and PAL predicted protest participation, while higher anxiety predicted non-participation.

**Conclusion:**

Findings highlight the psychological toll of democratic backsliding and identify political grief as a distinct emotional response linking political ambiguous loss to public mental health. Collective grief functions both as a psychological burden and as a mobilizing force. Recognizing such grief at the national level and implementing community-based mental health interventions are crucial to supporting populations during political crises.

## Introduction

In recent years, numerous societies have faced significant political and social upheavals, including shifts toward authoritarian governance (1). These transitions have evoked profound collective psychological reactions among citizens, prompting widespread protests and demonstrations globally (2, 3). Notable examples include Poland’s response to judicial reforms (4), Hong Kong’s pro-democracy movement (5, 6), and the worldwide impact of the Black Lives Matter movement (7).

Democracy, as conceptualized in both institutional and civic terms, refers not only to a system of governance sustained by the rule of law, separation of powers, and public participation, but also, as Dewey (8) emphasized, to a moral and educational mode of associated living that nurtures dialogue, equality, and civic responsibility. “Democratic backsliding” denotes the gradual, state-led erosion of these institutions and norms, typically enacted through legalistic and incremental means rather than overt coups (1, 9, 10). As Bermeo (9) underscores, contemporary declines in democracy often occur subtly and legally, under the guise of democratic legitimacy. Within this dual conception, what citizens experience as loss is twofold: the weakening of institutional safeguards that ensure accountability and rights, and the disruption of democracy as a shared way of life grounded in mutual trust, civic engagement, and collective moral ethos.

Protracted political instability and threats to democracy significantly impact public mental health, consistently triggering negative emotions such as stress, depression, and anxiety (6, 11, 12). These emotions, exacerbated by continuous uncertainty surrounding sustained protests, have been linked to worsened psychological and physical well-being (11). A review by Ni et al. (13) underscores that individuals living in protest-affected regions, particularly those marked by violence, frequently exhibit elevated rates of Post-Traumatic Stress Disorder (PTSD), anxiety, and depression. A recent study by Levi-Belz et al. (14) found high distress levels among Israeli protesters, with 44.6% meeting criteria for major depression and 10.6% for probable PTSD.

Alongside psychological distress, perceived oppression and threats to democratic institutions can trigger collective grief, especially when communities sense their core societal values are being undermined (15). Eisenbruch (16) highlights that grief, shaped by cultural meaning systems, may evolve into grievance—a collective emotional response fueling political engagement and mobilization aimed at restoring justice, freedom, or national ethos.

Harris (17) conceptualizes this emotional phenomenon as *political grief—a* profound response to the perceived erosion of values, ideology, and identity resulting from disruptions to individuals’ “assumptive world” (18), the internal framework that provides stability, security, and predictability. In Bowlby (19) attachment theory terms, such political loss can shatter citizens’ internal working models—the mental representations that organize expectations about the self, others, and the world. Harris (17) illustrates political grief through reactions to Donald Trump’s 2016 election victory, when supporters of Hillary Clinton experienced collective grief over perceived losses in national identity and democratic norms (20, 21). From this perspective, civic activism and protest can be viewed as efforts to restore a ruptured attachment and reassert psychological coherence. Grief, in this sense, is an adaptive process through which individuals reconcile the world as it now exists with the world they once believed to exist (17). However, little is known about the course and nature of grief in an unwanted yet potentially reversible political reality (loss).

Such experiences of slow and gradual backsliding align with the concept of *ambiguous loss* (22), traditionally applied to personal relationships in which a loved one’s physical or psychological presence is uncertain. More recently, ambiguous loss has been extended to collective contexts such as immigration (23) and the COVID-19 pandemic (24, 25). In political contexts, ambiguous loss may arise when a country’s ideological identity becomes unstable, creating a profound sense of disconnection among citizens who struggle with uncertainty about the nation’s future and their own sense of belonging and security (26). Especially in gradual and slow processes in which democratic backsliding occurs (9, 10), political loss can be ambiguous and ongoing in nature. These conditions are known to elicit grief and separation-distress symptoms that are inherently prolonged, as citizens oscillate between hope for return and despair (27–29). Moreover, unlike bereavement following death, these non-death losses lack closure, clear rituals, or public recognition, thereby disenfranchising grief and intensifying distress (30).

Hope et al. (12) demonstrated that political activism may serve as a coping mechanism for marginalized youth, helping to buffer the psychological impact of discrimination and distress. Similarly, Ford et al. (11) found that daily political events consistently evoked negative emotions, which were associated with reduced well-being but also increased political action, such as volunteering and protesting. However, while much research focuses on psychological distress (e.g., anxiety, depression) in response to political instability, fewer studies have examined how complex emotional experiences such as political grief and ambiguous loss contribute to distress. Moreover, the precise relationship between these experiences and political engagement remains understudied, particularly regarding how specific emotional states and personal traits impact individuals’ decisions to engage in activism.

One relevant personal trait in this context is political efficacy, the belief in one’s ability to influence political processes (31, 32). Political efficacy comprises external efficacy, belief in system responsiveness, and internal efficacy—confidence in one’s political competence (31, 33, 34). Higher political efficacy may buffer mental health adversities; activists with stronger efficacy beliefs report fewer anxiety, depression, and PTSD symptoms despite experiencing significant trauma (35, 36). In scenarios of democratic erosion, efficacy beliefs significantly influence whether citizens choose activism or resignation, reflecting their sense of civic duty or perceived voice efficacy.

Taken together, democratic backsliding can be a macro-level stressor that destabilizes citizens’ assumptive world, evoking political ambiguous loss characterized by uncertainty, indeterminacy, and lack of resolution. Such ambiguous loss gives rise to inherently prolonged political grief (i.e., grief-like emotional and cognitive responses directed at perceived political loss), which, in unresolved non-death contexts, co-occurs with heightened distress. This heightened emotional state, encompassing both grief and distress, may in turn motivate civic engagement. Political efficacy functions as an individual resource shaping whether this distress and grief translate into action (e.g., protest participation). Together, these processes—backsliding → ambiguous loss → political grief → distress and engagement—form the theoretical foundation for the current study.

### The present study

In early 2023, Israel’s newly formed right-wing coalition government swiftly advanced a sweeping plan to overhaul the judiciary, which many scholars described as one of the most far-reaching institutional changes in the country’s history. The proposed reforms aimed to curtail the Supreme Court’s authority to review legislation, significantly alter the composition and appointment process of judges, and reduce legal oversight of executive actions. Legal scholars warned that these measures threatened the separation of powers and the independence of the judiciary, key pillars of liberal democracy, and thus constituted a process of democratic backsliding through institutional erosion (10, 37, 38). In reaction, the country experienced one of the most prolonged protest movements in its history, with mass demonstrations, weekly rallies, and civil resistance actions sustained for 39 weeks, until the outbreak of war on October 7. This unprecedented civic mobilization reflected deep public opposition to the perceived threat posed to democratic norms.

The present study, conducted in August 2023 (weeks 33–36 of the nationwide protests), sought to investigate the impact of democratic backsliding on the mental health of Israeli citizens opposing the judicial reforms, with a focus on psychological distress, political ambiguous loss, and political grief, as well as the factors predicting civic engagement in protest. Specifically, the study aimed to: (1) assess the prevalence of psychological distress (depression, anxiety, and stress) and political grief among citizens opposing the judicial reform; (2) examine the relationship between political ambiguous loss and psychological distress, with political grief as a potential mediator; and (3) investigate how emotional states (psychological distress, political grief, and political ambiguous loss) and personal traits (political efficacy) predict individuals’ decisions to engage in activism.

We hypothesized: (1) Political ambiguous loss would be associated with greater psychological distress, mediated by political grief; and (2) Political efficacy, political ambiguous loss, political grief and psychological distress were expected to positively predict protest participation. To our knowledge, this is the first empirical study to examine political ambiguous loss and grief in the context of democratic backsliding and prolonged civic resistance.

## Methods

### Participants and procedure

This cross-sectional study included a convenience sample of 453 Israeli citizens aged 18 or older who self-identified as opposing the 2023 judicial reform. Participants were recruited in August 2023 through online platforms (e.g., social media groups, digital forums) affiliated with Israel’s civil protest movement. The recruitment post stated: “We invite Israeli citizens aged 18 and above to take part in a short anonymous study examining emotional and psychological responses to the judicial reform.” Individuals could access the survey via a secure Qualtrics link after reading full study information and providing electronic consent. This purposive sampling strategy was designed to examine psychological and civic processes within a politically engaged population. Sample characteristics are presented in **Table 1**. The study received ethical approval from the Institutional Review Board (IRB) of The Academic College of Tel Aviv—Yaffo. Participation was voluntary, anonymous, and could be withdrawn at any time.

**Table 1:** Sample sociodemographic characteristics.

Variable	Levels	% (n), M (SD)
Gender	Man	36.2% (164)
	Woman	63.8% (289)
Age		51.53 (14.00)
Education	Elementary	0.4% (2)
	High school	2.6% (12)
	Certification studies	5.3% (24)
	B.A.	26.5% (120)
	M.A.	47.9% (217)
	PhD	17.2% (78)
Family status	Single	11.3% (51)
	Living together	15.9% (72)
	Married	63.8% (289)
	Separate	1.1% (5)
	Divorced	5.3% (24)
	Widowed	2.6% (12)
Socio-economic status	Far below average	4.4% (20)
	Below average	9.7% (44)
	Average	15.5% (70)
	Above average	51.4% (233)
	High above average	19.0% (86)
Number of prior traumatic life events		0.59 (0.73)
Number of psychiatric diagnoses		0.38 (0.68)

The survey was administered in a fixed order. Participants first completed sociodemographic items, the checklist of prior life events, and the checklist of prior psychiatric disorders, followed by the protest participation item. Among the psychological measures, the psychological distress scale was presented first, preceding the items related to political grief, political self-efficacy and political ambiguous loss.

### Measures

#### Psychological distress

The Depression Anxiety Stress Scales–21 (DASS-21) (39) was used to measure participants’ psychological distress. This 21-item self-report instrument assesses three interrelated dimensions of emotional distress: *depression* (e.g., low mood, loss of self-esteem, and anhedonia), *anxiety* (e.g., fear, physiological hyperarousal, and anticipation of negative events), and *stress* (e.g., persistent tension, irritability, and low frustration tolerance). Participants were asked to assess each of the 21 items, seven per subscale, on a four-point Likert scale, ranging from 0 (“did not apply to me at all”) to 3 (“applied to me very much, or most of the time”), based on the past week. Higher scores reflect greater emotional distress. Items included statements such as “I found it hard to wind down,” “I was aware of dryness of my mouth,” and “I couldn’t seem to experience any positive feeling at all.” Results were interpreted according to the subscale cut-offs (40), which categorize levels of distress from “normal” to “extremely severe”.

The Hebrew version, obtained from the National Psychology Unit of the Israeli Ministry of Health, demonstrated good reliability in the current sample: DASS anxiety (ω = 0.74), DASS depression (ω = 0.86), DASS stress (ω = 0.87), and overall DASS score (ω = 0.92).

#### Political grief

An adapted version of the Prolonged Grief Disorder Questionnaire (PG-13) (41)—Hebrew version (42)—was used to assess participants’ grief responses to political loss. The PG-13, originally developed as a diagnostic instrument, comprises 11 items assessing cognitive, emotional, and behavioral symptoms on a 5-point Likert scale (1 = not at all to 5 = several times a day/almost always). These are followed by two yes/no items assessing symptom duration (over six months) and functional impairment, in accordance with DSM-5 criteria. Consistent with previous adaptations of the PG-13 to symbolic, non-death losses such as divorce and unemployment (43, 44), the scale assessed participants’ emotional reactions to perceived deterioration in the state of the country with respect to the announced judicial reform. Sample adapted items include: “In the past month, how often have you had intense feelings of emotional pain, sorrow, or grief related to the losses resulting from the changes occurring in the country?”; “In the past month, how often have you tried to avoid reminders that the country is no longer as it once was?”. The full adapted instrument is provided in **Supplementary Materials 1**: Adapted PG-13 for Political Loss (English and Hebrew Versions). A total score was calculated by summing responses on the 11 symptom items, yielding a range of 11 to 55, with higher scores indicating greater symptom severity. The scale demonstrated high internal reliability in the current sample (Ω = 0.84), consistent with previous findings (41, α = 0.82).

*Cut-off interpretation.* We reported proportions above the original threshold as descriptive indicators of elevated grief-like severity, not diagnostic classifications. Given that this study addresses an ongoing, collective, and ambiguous loss rather than bereavement, these cut-offs serve only to contextualize relative symptom intensity. For consistency with prior research on the original instrument, we applied the original ≥30 threshold (41) and a conservative ≥35 threshold validated in clinical studies (42, 45, 46).

#### Political efficacy

The Internal and External Political Efficacy Questionnaire (31, 33, 34) was used to assess participants’ political efficacy. This combined measure was developed by Scotto et al. (31) based on earlier foundational work (33, 34). It includes six items assessing internal efficacy and four items assessing external efficacy, rated on a six-point Likert scale (1 = strongly disagree to 5 = strongly agree, with a “don’t know” option coded as missing). Internal efficacy refers to beliefs about one’s competence to understand and participate effectively in politics, while external efficacy reflects beliefs about the responsiveness of governmental authorities and institutions to citizen demands. Sample items include: “I think that I am better informed about politics and government than other people” (internal efficacy) and “People like me don’t have any say about what the government does” (external efficacy). Mean scores range from 1 to 5, with higher scores indicating greater perceived efficacy. While the combined scale’s reliability has not been formally established, the item set has demonstrated cross-cultural validity in measuring political efficacy outside the U.S. context (31, 32). In the current study, the items were translated into Hebrew using the back-translation method. The internal reliability of the scale in this sample was good (ω = 0.85).

#### Political ambiguous loss scale

The scale was developed specifically for this study to assess the emotional ambiguity and uncertainty experienced by Israeli citizens in response to recent political changes. The scale’s development was guided by two key theoretical frameworks. First, it draws on Boss (22) concept of ambiguous loss to capture the profound uncertainty and lack of closure. Second, it is grounded in Harris (17) definition of *political loss* to define the *content* of that loss. As Harris (17) articulates, this form of loss is a complex phenomenon that explicitly includes a “sense of paralysis that occurs as a result of the deep divisions and polarizations” and is directly equated with a “loss of identity” and a “loss of predictability and safety.” Six items were generated by two experts in ambiguous loss to reflect the intersection of these theoretical frameworks with public discourse, including shattered assumptions, national uncertainty, loss of security, and disrupted political identity (e.g., “I am unsure about the future of my country in light of the emerging political change”). Items were rated on a 5-point Likert scale (1 = strongly disagree to 5 = strongly agree), with total scores ranging from 6 to 30. Higher scores indicate greater emotional unease and ambiguity in relation to one’s connection with the state. The scale demonstrated good internal consistency (ω = 0.84) and a unidimensional structure based on exploratory factor analysis, with all items loading ≥.54 and 47.4% of variance explained (see **Appendix 1**).

#### Protest participation

Participation in the protest was assessed using a two-step item designed to yield a binary outcome (active participation = 1; no participation = 0) and address the potential ambiguity of what constitutes “protest.” Participants first indicated whether they were involved in the protest against the judicial reform (“Are you involved in the protest?”; yes/no). Those who responded “yes” were then presented with a multiple-choice, multiple-response question asking, “What is the nature of your involvement?” and were asked to select all that apply from a list of activities. The options provided were: “Writing posts on social media,” “Attending a demonstration in my residential area,” “Attending an organized demonstration far from my residential area,” “Organizing demonstrations and protest groups,” and “Other (please specify).” For the analysis, participants were considered “active” and coded as ‘1’ if they responded “yes” to the first question and selected at least one specific activity from the list. Participants who only selected “Other” (2.5% of the sample) had their responses manually reviewed; all self-described activities (e.g., “donation,” “organizing debates,” “organizing and signing petition”) were assessed as active involvement, and these participants were thus coded as ‘1’.

#### Sociodemographic and background variables

This section collected data on participants’ age, gender, socioeconomic status (SES), family status, and educational level. SES was assessed using a single item asking participants to rate their household income relative to the national average on a 5-point scale (from 1 = “Far below average” to 5 = “High above average”).

Participants’ exposure to *prior difficult/traumatic life events* was also assessed using a checklist. The item asked participants to select all events they had experienced from a list (e.g., immigration, death of a loved one, job loss). The list also included an “Other traumatic event (please specify)” option and a “None of the above” option. For the analysis, a variable was created by summing the total number of events endorsed by each participant (with “None of the above” coded as 0). This variable is referred to as ‘Number of prior traumatic life events’ in the analyses.

Finally, participants were asked to self-report any *prior psychiatric diagnoses* they had ever received from a mental health professional. This was presented as a checklist of common disorders, from which participants could select all that applied. The list also included a “None of the above” option. For the analysis, a variable was created by summing the total number of disorders endorsed by each participant (with “None of the above” coded as 0). This variable is referred to as ‘Number of psychiatric disorders’ in the analyses.

### Data analysis

All analyses were conducted using R version 4.3.0 (47). To address 4.6% missing data on the protest involvement duration variable, multiple imputation was performed using the *mice* package (48), generating 21 imputed datasets. An exploratory factor analysis (EFA) was conducted to assess the structure of the political ambiguous loss scale; detailed results of this analysis are presented in **Appendix A**. Descriptive statistics were computed for psychological distress (DASS-21) and political grief-like symptom intensity (adapted PG-13). For the latter, proportions above established PG-13 thresholds were reported as descriptive indicators of elevated political grief severity. Pearson correlations between demographic variables and the primary study variables were also calculated. To test the hypothesized mediation model, we conducted a path analysis using structural equation modeling (SEM) with the *lavaan* package (49), employing a bootstrap procedure with 5,000 random samples. All independent variables were standardized prior to entry into the SEM model. In each model, we controlled for socio-demographic variables which were associated with the mediating variable (political grief) or each of the dependent variables (DASS-21 subscales): gender, SES, number of prior psychiatric disorders, and number of prior life events. Notably, although education had no significant correlation with the mediating or dependent variables, it was entered as a control due to its distribution in the sample. Finally, a hierarchical multivariate logistic regression analysis was performed to predict protest participation. The analysis progressed through four sequential models: Model 1 included demographic variables; Model 2 added political efficacy; Model 3 introduced psychological distress variables (DASS-Stress, DASS-Anxiety, and DASS-Depression); and Model 4 incorporated political ambiguous loss and political grief (adapted PG-13) scores as predictors.

## Results

### Descriptive analysis: prevalence of mental health symptoms

As shown in **Table 2**, 32.0% of participants reported moderate to severe symptoms of depression, 10.8% reported moderate to severe anxiety, and 26.3% reported moderate to severe stress. Additionally, 64.9% of the sample scored above the established PG-13 threshold, indicating elevated political grief severity.

**Table 2:** Distribution of psychological distress (DASS-21) and political grief (PG-13) scores in the sample.

Variable	% Normal (n)	% Mild (n)	% Moderate (n)	% Severe (n)	% Extremely severe (n)
DASS depression	49.7% (225)	18.3% (83)	21.4% (97)	7.7% (35)	2.9% (13)
DASS anxiety	76.6% (347)	12.6% (57)	6.2% (28)	2.6% (12)	2.0% (9)
DASS stress	59.6% (270)	14.1% (64)	16.6% (75)	7.3% (33)	2.4% (11)
Political Grief (Adapted PG-13)		proportion above threshold ≥30, 64.9% (n = 294)			
Political Grief (Adapted PG-13)		proportion above threshold ≥35, 36.2% (n = 164)			

DASS, *Depression, Anxiety, and Stress Scales*; *PG-13, Prolonged Grief-13 scale;* Proportions above thresholds are reported as descriptive indicators of elevated political grief severity, not as diagnostic classifications.

### Correlations between study variables and demographic characteristics

Pearson correlation analyses revealed several significant associations between the study variables and demographic characteristics. Political efficacy was negatively associated with gender (*r* = −0.20, *p* < 0.001), suggesting that men reported higher levels of efficacy than women, and positively associated with both age (*r* = 0.23, *p* < 0.001) and SES (*r* = 0.21, *p* < 0.001). Political ambiguous loss scores were significantly associated with gender (*r* = 0.13, *p* = 0.007), indicating that women reported higher levels of political ambiguous loss. Political grief, as measured by adapted PG-13 scores, was positively correlated with the number of prior traumatic life events (*r* = 0.09, *p* = 0.048) and the number of psychiatric disorders (*r* = 0.12, *p* = 0.013).

Regarding psychological distress, DASS-depression scores were negatively associated with age (*r* = −0.20, *p* < 0.001) and SES (*r* = −0.17, *p* < 0.001) and positively associated with the number of psychiatric disorders (*r* = 0.22, *p* < 0.001). DASS-anxiety scores were positively correlated with gender (*r* = 0.19, *p* < 0.001), the number of prior traumatic life events (*r* = 0.11, *p* = 0.019), and the number of psychiatric disorders (*r* = 0.20, *p* < 0.001), and negatively correlated with age (*r* = −0.25, *p* < 0.001) and SES (*r* = −0.21, *p* < 0.001). DASS-stress scores were significantly associated with gender (*r* = 0.16, *p* < 0.001), age (*r* = −0.18, *p* < 0.001), and number of psychiatric disorders (*r* = 0.15, *p* = 0.001). No other significant correlations were found between gender, age, SES, number of prior traumatic life events, education and the remaining study variables. The complete correlation matrix of the study variables is presented in **Table 3**.

**Table 3:** Descriptive and correlation analysis for study variables.

Variable	M (SD)	1	2	3	4	5	6	7
1. Political efficacy	3.38 (0.59)							
2. Protest participation^a^	0.82 (0.38)	0.22^***^						
3. Involvement duration	26.32 (6.76)	0.32^***^	–					
4. Political Ambiguous Loss (PAL)	3.70 (0.84)	−0.03	0.19^***^	0.14^**^				
5. Political Grief (Adapted PG-13)	31.96 (7.41)	−0.02	0.15^**^	0.13^*^	0.70^***^			
6. DASS—depression	5.18 (3.93)	−0.16^***^	0.05	0.01	0.41^***^	0.56^***^		
7. DASS—anxiety	2.16 (2.58)	−0.19^***^	−0.03	−0.14^**^	0.23^***^	0.39^***^	0.63^***^	
8. DASS—stress	7.00 (4.23)	−0.06	0.13^**^	−0.01	0.41	0.54^***^	0.71^***^	0.63^***^

a 0 = not involved, 1 = involved; DASS = Depression, Anxiety and Stress Scale; PG-13 = Prolonged Grief-13 scale. ^*^p < 0.05; ^**^p < 0.01; ^***^p < 0.001

### The role of political grief in the relationship between political ambiguous loss and psychological distress: mediation analysis

**Figure 1:**
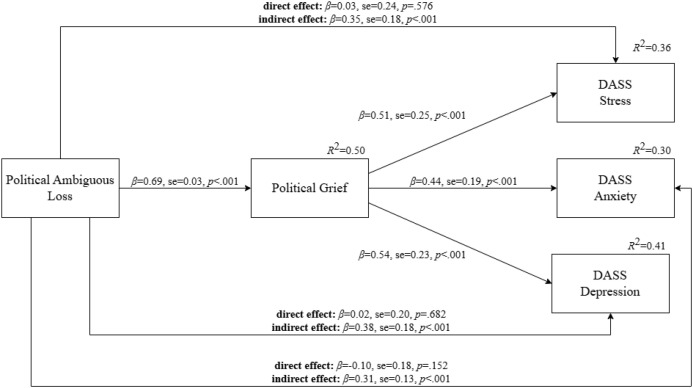
Political grief as a mediator between political ambiguous loss and psychological distress (depression, anxiety, and stress). Standardized coefficients are shown; DASS, Depression, Anxiety, and Stress scale.

**Figure 1** presents the results of the mediation model examining the role of grief in the relationship between political ambiguous loss and psychological distress (depression, anxiety, and stress). Relevant covariates were included for each outcome: the number of prior traumatic events and psychiatric diagnoses were controlled for in the political grief model; age, SES, and psychiatric diagnoses were controlled for in the DASS-depression model; gender, age, and psychiatric diagnoses in the DASS- stress model; and gender, age, SES, number of prior traumatic events, and psychiatric diagnoses in the DASS-anxiety model. The model demonstrated excellent fit: χ²(8) = 7.21, χ²/df = 0.90, *p* = .515, CFI = 1.00, RMSEA <.01. Political grief fully mediated the relationship between political ambiguous loss and all three DASS subscales—depression, stress, and anxiety. Additionally, there was no indication for multicollinearity, as the maximum VIF value was 2.02.

### Modeling protest participation: a multivariate logistic regression analysis

**Table 4** presents the results of the hierarchical multivariate logistic regression analysis predicting participation in the protest. In the first model, demographic variables were entered, only number of psychiatric disorders and level of education were significant predictors of protest participation so participants with higher number of psychiatric disorders and higher level of education were more likely to participate in the protest (Nagelkerke *R²* = 0.07, χ²(6) = 20.57, *p* = .002). In the second model, political efficacy was added and showed a significant contribution to the model (Nagelkerke *R*² = 0.14, χ²(1) = 21.11, *p* <.001), indicating that participants with higher political efficacy were significantly more likely to participate in the protest. The third model included DASS stress, anxiety, and depression scores (Nagelkerke *R*² = 0.19, χ²(3) = 14.72, *p* = .002). In this model, both stress and anxiety significantly predicted participation, but in opposite directions: higher stress was associated with increased likelihood of participation, while higher anxiety was associated with decreased likelihood. Depression scores did not significantly predict protest involvement. In the final model, political ambiguous loss and political grief (adapted PG-13) scores were added (Nagelkerke *R*² = 0.22, χ²(2) = 7.92, *p* = .019). Higher political ambiguous loss scores significantly predicted protest participation, whereas political grief did not show a significant effect. There was no indication for multicollinearity, as the maximum VIF value was 2.80.

**Table 4:** Multivariate logistic regression analysis predicting protest participation.

Predictors	Model 1		Model 2		Model 3		Model 4	
	OR	p	OR	p	OR	p	OR	p
Intercept	4.85	<.001	4.66	<.001	5.32	<.001	5.79	<.001
Gender^a^	1.07	.793	1.35	.287	1.29	.391	1.19	.559
Age	1.13	.333	1.03	.846	1.03	.843	1.01	.922
Socio-economic status	1.02	.894	0.92	.544	0.88	.394	0.83	.217
Number of prior traumatic life events	1.08	.546	1.08	.576	1.10	.515	1.09	.558
Number of psychiatric disorders	1.40	.038	1.38	.049	1.41	.043	1.42	.043
Education	1.59	<.001	1.57	.001	1.54	001	1.54	.001
Political Efficacy			1.84	<.001	1.83	<.001	1.84	<.001
DASS—Depression					0.99	.969	0.84	.424
DASS—Anxiety					0.61	.009	0.66	.027
DASS—Stress					2.05	.001	1.81	.012
Political Ambiguous Loss							1.44	.047
Political Grief (Adapted PG-13)							1.07	.752
Negelekerke R^2^	0.07		0.14		0.19		0.22	
	χ^2^(6)=20.57, p=.002		χ^2^(1)=21.11, p<.001		χ^2^(3)=14.72, p=.002		χ^2^(2)=7.92, p=.019	

a 0 = men, 1 = women; DASS=Depression, Anxiety, and Stress scale.

## Discussion

To the best of our knowledge, this is the first empirical investigation examining political grief and ambiguous loss within the context of judicial reform and democratic backsliding. Our findings provide an integrated perspective on how political upheaval can significantly affect individual and collective psychological states and consequently influence civic behavior.

### The impact of judicial reform on the mental health of opponents

More than half of the sample (64.9%) scored above the original PG-13 threshold (≥30), indicating elevated political grief severity, while 32.0% reported moderate to severe depression, 26.3% reported moderate to severe stress, and 10.8% reported moderate to severe anxiety. Using a more conservative PG-13 threshold (≥35), 36.2% of participants still exhibited high levels of grief-related distress. Although the gap (64.9% *vs*. 36.2%) is an expected result of the more conservative threshold, the key finding remains: over one-third of the sample reported severe, prolonged political grief symptoms.

This pattern aligns with previous research linking political instability to psychological distress (6, 11, 50). It also accords with recent findings from Levi-Belz et al. (14), who reported that 44.6% met criteria for major depression and 10.6% for probable PTSD within a similar Israeli protest population. Although our observed depression rate (32.0%) was somewhat lower than their finding, it remains markedly higher than international protest samples, where depression rates typically average around 10% (13). These findings underscore a substantial mental health burden among Israeli citizens opposing the judicial reforms.

### The relationship between political ambiguous loss and psychological distress: the mediating role of political grief

The mediation model (political ambiguous loss → political grief → psychological distress) clarifies the mechanism through which political ambiguous loss translates into heightened psychological distress via political grief, accounting for a substantial portion of variance across outcomes, ranging from 30% to 41% in anxiety, stress, and depression, even after controlling for socio-demographic factors. This relationship illustrates how political uncertainty and a lack of clarity about the country’s future can intensify emotional suffering, enriching our understanding of the psychological consequences of political loss in the context of democratic backsliding.

Building on Bowlby’s attachment theory (51), we propose that the profound ambiguous loss associated with political upheaval triggers separation distress, manifesting in grief symptomatology. Citizens experience a psychological struggle akin to mourning as they attempt to reconcile their attachment to the nation amid its reconfigurations. Chan (26) highlighted how collective grief may transcend emotional distress and take on existential dimensions, as seen in Hong Kong residents grieving the symbolic loss of their hometown. This dynamic is especially relevant to the Israeli context, where democratic erosion undermines deeply held assumptions regarding security, justice, and civic identity (10, 17, 38), thereby amplifying psychological distress and moral injury (52, 53).

The erosion of trust, coupled with institutional tactics such as gaslighting, may further intensify grief and alienation (26, 52). Evidence from the COVID-19 pandemic also underscores the role of institutional trust in buffering against grief and worry, fostering resilience at both the personal and community levels (54). Consequently, this emotional turmoil may amplify psychological distress by contributing to a broader experience of collective political grief. While some have theorized that political grief can motivate civic action (7, 15, 55), our mediation model situates political grief primarily as an emotional response to political loss and its derivatives that exacerbates distress. This distinction will be revisited in the following section on protest participation.

### The role of emotional states and political efficacy in protest participation: a regression analysis

Our regression analysis indicated that higher levels of anxiety were found to predict non-participation, whereas higher levels of stress, political ambiguous loss, and political efficacy significantly predicted active involvement in protests. These results shed light on the emotional underpinnings of political activism, suggesting that while certain emotional states may immobilize individuals, others may serve as catalysts for action. This pattern aligns with prior findings. For example, Ford et al. (11), using diary studies, demonstrated that daily political events often elicit negative emotions, which are associated with diminished psychological well-being but increased political engagement—driven by a desire to respond to the very systems producing those emotions. Similarly, Chang et al. (6) identified stress as a significant motivator for political involvement, proposing that participation may function as a coping strategy during times of uncertainty. Other studies have highlighted the distinct motivational roles of specific emotions: anger and moral outrage tend to mobilize protest, whereas fear and hopelessness are more likely to inhibit participation (56–58).

In our study, the role of political efficacy aligned with previous findings highlighting its importance in predicting active political engagement. Individuals with higher perceived efficacy were more likely to participate in protests despite experiencing emotional distress (31, 33). Moreover, our correlational analysis indicated that higher political efficacy was associated with lower levels of anxiety and depression (see **Table 3**), echoing prior studies suggesting that political efficacy may buffer against mental health adversities (35, 36). During the 2023 protests in Israel, widespread participation was often framed in public discourse as a civic duty to defend democratic norms. However, our findings suggest that not all individuals responded to political distress with activism. In particular, individuals experiencing higher levels of anxiety were less likely to participate in protests, while those with higher levels of political efficacy were more likely to engage. This aligns with Baron et al. (59), who argued that protest participation involves weighing emotional responses, perceived threats, and beliefs in the effectiveness of collective action. In this context, perceived political efficacy appears to be a key factor enabling individuals to overcome emotional barriers and take action.

Lastly, when both political ambiguous loss and political grief were entered into the final regression model, only political ambiguous loss emerged as a significant predictor of protest participation. This finding is consistent with the mediation model’s structure, which positions the experience of political loss as the antecedent to the grief response (17, 19). It suggests that the core experience of political ambiguous loss—which, as defined in this study, captures not just uncertainty but also the perceived loss of identity, safety, and national cohesion stemming from deep political divisions (17)—may be a more direct motivator for civic action than the resulting grief symptoms alone. While collective grief is more emphasized in the context of responses to sociopolitical events (15, 16, 20, 21), rather than the underlying experience of loss itself, our findings highlight the importance of distinguishing between the two. Although the unique variance explained by political ambiguous loss in predicting protest participation was modest, its statistical significance and theoretical novelty point to the relevance of ambiguous loss as an emotional mechanism in political civic engagement.

Notably, these findings underscore the interplay between emotional states, personal traits, and civic action. While some emotions, such as stress, may motivate protest participation, paradoxically, sustained involvement can strain mental health. Although protest intensity was not assessed in the current study, prior research suggests that prolonged activism may exacerbate psychological distress (13, 14). This point is strongly reinforced by Hamama-Raz et al. (60), who studied the same 2023 Israeli protest context and found that participants with *higher engagement* in the unrest reported *higher levels of distress and anxiety* compared to those with no engagement.

### Implications

The findings of this study carry several important implications. First, there is a pressing need for governmental and state institutions to acknowledge the psychological toll that political instability and democratic erosion impose on citizens. Such recognition can serve as public validation, helping to mitigate feelings of disenfranchisement and affirm the legitimacy of collective grief (30). In parallel, civic and mental health organizations should actively work to bring public attention to this emotional toll and advocate for official acknowledgment when it is absent. Framing emotional responses as reactions to a moral breach in the social contract may help restore trust between citizens and institutions.

Although not directly assessed in this study, collective actions such as protests may serve as grief rituals—offering communal spaces to mourn shared losses of democratic values and identity (7, 15, 24). These acts can simultaneously express loss and resistance, allowing communities to reaffirm their agency and vision of justice (16). In this sense, civic engagement may function as both a coping mechanism (61) and a reparative force. Therefore, civil society organizations, community leaders, and mental health practitioners should facilitate inclusive participation and community-based dialogue as protective psychological processes and vital expressions of democratic vitality, particularly when state institutions fail to do so.

Collective grief in political crises often activates historical trauma, perpetuates cycles of polarization, and exposes entire communities to secondary trauma and moral injury (13, 14). It may also escalate into existential crises, leading to intangible losses such as trust, belonging, and social cohesion (24, 25, 62). When state responses are perceived as inadequate, they risk deepening public distress, mistrust, and alienation (54). To interrupt these cycles and promote societal resilience, international human rights organizations, policy think tanks, and future pro-democracy administrations should work to address the sociopolitical roots of collective grief by fostering public dialogue, transparency, and trust.

The high rates of psychological distress and political grief observed in this study reflect a significant emotional burden among citizens opposing the judicial reform. These findings highlight the psychological toll of sustained political crisis and underscore the urgent need for community-based mental health interventions supported by both governmental and non-governmental sectors. Such interventions- like civic dialogue, grief-informed education, and accessible psychosocial services—can foster collective resilience and restore a sense of social coherence. Importantly, political efficacy emerged as a protective factor, associated with both increased civic engagement and lower psychological distress. Practitioners, educators, and NGOs can play a key role in promoting efficacy by strengthening citizens’ sense of agency, voice, and influence, thereby serving as a psychological buffer in times of democratic backsliding and political uncertainty.

### Limitations and future directions

This study provides rare empirical evidence of collective political grief and ambiguous loss, underscoring their relevance as public mental health concerns during democratic backsliding. Nonetheless, the findings must be interpreted in light of several limitations. Its cross-sectional design prevents causal inference, and the mediation analysis should be interpreted as exploratory. The sample was a non-representative convenience sample, drawn from a politically engaged population opposing the judicial reform. As such, the elevated levels of psychological distress and political grief observed cannot be generalized to the broader Israeli public. However, they do underscore the psychological vulnerability of individuals who experience these events as a form of democratic loss.

The sample’s characteristics also present limitations. The sample was highly educated (65% held M.A. or Ph.D. degrees), which may reflect a socio-economically advantaged, civically active group. Although we controlled for education, how class and education shape political grief requires further investigation. Furthermore, recruitment through protest-affiliated networks introduced self-selection bias. While this limits representativeness, it provided valuable insight into the psychological experiences of citizens most directly involved in the crisis.

Several measurement limitations should also be acknowledged. Although prior exposure to life events was controlled, participants’ political engagement before the reform was not assessed and may have influenced current distress and activism. Political efficacy was also treated as a stable trait, though evidence suggests it can be shaped by participation itself (63). Protest participation was measured as a binary outcome, limiting our ability to assess its intensity, frequency, or subjective meaning. Furthermore, although we used established PG-13 scoring to contextualize prolonged grief severity, future research should establish validated cut-off scores for political grief specifically as well as directly validate adapted PG-13 questionnaire (e.g. CFA, test-retest reliability, convergent and discriminant validity). Future studies should also move beyond bereavement-based models to better capture the ongoing and unresolved nature of political grief, and more research is needed to distinguish between adaptive versus maladaptive grief trajectories within these specific political contexts.

The political ambiguous loss scale used in this study was exploratory and not fully validated psychometrically. In addition, we did not include direct measures of attitudes toward democracy, perceived democratic erosion, or political polarization, which may serve as additional contextual triggers of political grief. Future research should work to disentangle these overlapping sources of distress associated with political loss (e.g., polarization-induced loss, societal unrest) by incorporating such measures and by comparing populations with differing political orientations (e.g., reform supporters). Future research should refine and adapt this measure to other sociopolitical contexts to examine its broader applicability and potential as a framework for understanding collective responses to political upheaval.

## Data Availability

The raw data supporting the conclusions of this article will be made available by the authors, without undue reservation.

## Appendix 1

Political Ambiguous Loss Scale: Item Wording, Descriptive Statistics, Factor Loadings from Exploratory Factor Analysis, and Internal Consistency (ω)

**Table:**

Item	Factor 1
1. The change in my country’s political fabric has negatively affected my sense of security.	0.80
2. I am uncertain about my country’s future in light of the emerging political change.	0.78
3. I feel disconnected from the political system, which is evolving in an unfamiliar or unknown direction.	0.58
4. The current political situation has led me to reconsider my identity as a citizen of the state.	0.54
5. I am worried that the country will become unrecognizable due to the emerging political change.	0.74
6. The prevailing ambiguity surrounding the country's future causes me psychological distress.	0.64
Percent explained variance	47.40%
Coefficient Ω for scale	0.84
Scale mean	3.70
Scale SD	0.84
