# Supplementary material for: Political grief and ambiguous loss in a threatened democracy: psychological distress and civic responses during Israel’s judicial reform
Source: Front Psychiatry. 2026 Jan 12;16:1687951. doi: 10.3389/fpsyt.2025.1687951 (PMC12834130; doi:10.3389/fpsyt.2025.1687951)
Supplement: Supplementary file 1 [file DataSheet1.pdf]

This instrument is an original adaptation developed by Dr. Einat Yehene. Use, reproduction, distribution, or adaptation of this instrument in any form is prohibited without prior written permission from the author. Copyright © 2025 by Einat Yehene.

Supplementary Material 1

Adapted Prolonged Grief Disorder Scale (PG-13; Prigerson et al., 2009) for Political Loss
(English and Hebrew Versions)

Caption. This supplementary file includes the adapted version of the Prolonged Grief Disorder Scale (PG-13) tailored to assess political grief in the context of the 2023 Israeli judicial reform. Both English and Hebrew versions are provided.

Political Grief Scale (Adapted Version, 2025)

Author: Dr. Einat Yehene
Affiliation: School of Behavioral Sciences, The Academic College of Tel Aviv–Yaffo, Israel
Contact: yeheneei@mta.ac.il

Recommended citation for this file:
Yehene, E. (2025). Political Grief Scale (Adapted Version) [Supplementary material]. In Yehene, E., & Ohayon, S. (2025). Political Grief and Ambiguous Loss in a Threatened Democracy: Psychological Distress and Civic Responses during Israel's Judicial Reform. *Frontiers in Psychiatry.* https://doi.org/10.3389/fpsyt.2025.1687951

English Version

Instructions
In responding to the following items, please refer to the changes in the country as you have experienced them since the announcement of the judicial reform (January 2023).
There are no right or wrong answers. Please try to respond to each question, even if you are uncertain of your answer.

Part A

For each item, mark the answer that best describes how you have felt **during the past month**.

1. In the past month, how often have you felt yourself longing or yearning for the country as you experienced it before the changes?
   1 = Not at all   2 = At least once   3 = At least once a week   4 = At least once a day
   5 = Several times a day

2. In the past month, how often have you had intense feelings of emotional pain, sorrow, or grief related to the losses resulting from the changes occurring in the country?
   1 = Not at all   2 = At least once   3 = At least once a week   4 = At least once a day
   5 = Several times a day

This instrument is an original adaptation developed by Dr. Einat Yehene. Use, reproduction, distribution, or adaptation of this instrument in any form is prohibited without prior written permission from the author. Copyright © 2025 by Einat Yehene.

3. For questions 1 or 2 above, have you experienced either of these feelings at least daily and after six months have elapsed since the change?
   ☐ No ☐ Yes

4. In the past month, how often have you tried to avoid reminders that the country is no longer as it once was?
   1 = Not at all 2 = At least once 3 = At least once a week 4 = At least once a day 5 = Several times a day

5. In the past month, how often have you felt stunned or shocked by the changes that have taken place in the country?
   1 = Not at all 2 = At least once 3 = At least once a week 4 = At least once a day 5 = Several times a day

Part B

For each item, please indicate how you feel **at present**.

6. Do you feel confused about your role in life or feel as if you don't know who you are (for example, a sense that a part of yourself has died)?
   1 = Not at all 2 = A little 3 = Somewhat 4 = Quite a bit 5 = Extremely

7. Have you had trouble accepting the changes that have occurred in the country?
   1 = Not at all 2 = A little 3 = Somewhat 4 = Quite a bit 5 = Extremely

8. Has it been hard for you to trust other people since these changes took place?
   1 = Not at all 2 = A little 3 = Somewhat 4 = Quite a bit 5 = Extremely

9. Do you feel bitter about the changes that have taken place in the country?
   1 = Not at all 2 = A little 3 = Somewhat 4 = Quite a bit 5 = Extremely

10. Do you feel that it would be difficult for you now to move forward with your life (for example, to make new friends or pursue new interests)?
    1 = Not at all 2 = A little 3 = Somewhat 4 = Quite a bit 5 = Extremely

11. Do you feel emotionally numb since the changes in the country?
    1 = Not at all 2 = A little 3 = Somewhat 4 = Quite a bit 5 = Extremely

12. Do you feel that your life has become unfulfilling, empty, or meaningless since the changes in the country?
    1 = Not at all 2 = A little 3 = Somewhat 4 = Quite a bit 5 = Extremely

Part C

13. Have you experienced a significant reduction in your ability to function in important areas of life—such as social, occupational, or family responsibilities?
    ☐ No ☐ Yes

This instrument is an original adaptation developed by Dr. Einat Yehene. Use, reproduction, distribution, or adaptation of this instrument in any form is prohibited without prior written permission from the author. Copyright © 2025 by Einat Yehene.

נספח A – גרסא עברית של שאלון האבל הממושך (PG-13 - Prigerson et al., 2009) מותאם לאובדן פוליטי.

הערה. הסולם עבר התאמה בהקשר לרפורמה המשפטית בישראל ב-2023.

Hebrew Original

הנחיות:
במענה לשאלות הבאות התייחס לשינויים שחלו במדינה כפי שאתה חווה אותם מאז ההכרזה על הרפורמה המשפטית (ינואר 2023).
אין תשובות נכונות או שגויות. אנא השתדל להשיב על כל שאלה, גם אם אינך בטוח בתשובתך.

חלק א'

לגבי כל פריט, סמן את התשובה המתארת בצורה הטובה ביותר כיצד הרגשת במהלך **החודש האחרון**.

1. במהלך החודש האחרון, באיזו תדירות חשת געגוע או כמיהה למדינה כפי שחווית אותה בעבר, לפני השינויים?
1 = כלל לא 2 = לפחות פעם אחת 3 = לפחות פעם בשבוע 4 = לפחות פעם ביום 5 = כמה פעמים ביום

2. במהלך החודש האחרון, באיזו תדירות חשת רגשות עזים של כאב רגשי, צער או אבל הקשורים לאובדנים הנובעים מהשינויים שחלו במדינה?
1 = כלל לא 2 = לפחות פעם אחת 3 = לפחות פעם בשבוע 4 = לפחות פעם ביום 5 = כמה פעמים ביום

3. בהתייחס לשאלות 1 או 2 לעיל, האם חווית אחת מהתופעות הנ"ל לפחות פעם ביום, ולאחר שחלפו שישה חודשים מאז השינוי?
☐ לא ☐ כן

4. במהלך החודש האחרון, באיזו תדירות ניסית להימנע מתזכורות לכך שהמדינה אינה כפי שהייתה?
1 = כלל לא 2 = לפחות פעם אחת 3 = לפחות פעם בשבוע 4 = לפחות פעם ביום 5 = כמה פעמים ביום

5. במהלך החודש האחרון, באיזו תדירות חשת המום או מזועזע מהשינוי שחל במדינה?
1 = כלל לא 2 = לפחות פעם אחת 3 = לפחות פעם בשבוע 4 = לפחות פעם ביום 5 = כמה פעמים ביום

חלק ב'

לגבי כל פריט, סמן את המספר המתאר כיצד אתה מרגיש **כעת**.

6. האם אתה מרגיש מבולבל בקשר לתפקידך בחיים, או כאילו אינך יודע מי אתה (למשל, תחושה שחלק ממך כבר מת)?
1= כלל לא 2 = במידה מועטה 3 = במידת מה 4 = די הרבה 5 = באופן גורף

This instrument is an original adaptation developed by Dr. Einat Yehene. Use, reproduction, distribution, or adaptation of this instrument in any form is prohibited without prior written permission from the author. Copyright © 2025 by Einat Yehene.

7. האם יש לך קושי להשלים עם השינוי שחל במדינה?
1= כלל לא 2 = במידה מועטה 3 = במידת מה 4 = די הרבה 5 = באופן גורף

8. האם קשה לך לבטוח באנשים מאז השינוי שחל במדינה?
1= כלל לא 2 = במידה מועטה 3 = במידת מה 4 = די הרבה 5 = באופן גורף

9. האם אתה מרגיש מר נפש בקשר לשינוי שחל במדינה?
1= כלל לא 2 = במידה מועטה 3 = במידת מה 4 = די הרבה 5 = באופן גורף

10. האם אתה מרגיש שקשה לך כעת להמשיך הלאה בחיים (כמו למשל לפגוש חברים חדשים או לקדם תחומי עניין חדשים)?
1= כלל לא 2 = במידה מועטה 3 = במידת מה 4 = די הרבה 5 = באופן גורף

11. האם אתה מרגיש קהה מבחינה רגשית מאז השינוי שחל במדינה?
1= כלל לא 2 = במידה מועטה 3 = במידת מה 4 = די הרבה 5 = באופן גורף

12. האם אתה מרגיש שהחיים שלך אינם מספקים, ריקים או חסרי משמעות מאז השינוי שחל במדינה?
1= כלל לא 2 = במידה מועטה 3 = במידת מה 4 = די הרבה 5 = באופן גורף

חלק ג'

13. האם חווית ירידה משמעותית ביכולתך לתפקד בתחומים חברתיים, תעסוקתיים או אחרים החשובים בחייך (כגון אחריות משפחתית או ביתית)?
☐ לא ☐ כן
